# Neoadjuvant therapy with chemotherapy and immune checkpoint inhibitor for laryngeal function preservation in locally advanced hypopharyngeal cancer

**DOI:** 10.3389/fimmu.2024.1364799

**Published:** 2024-03-08

**Authors:** San-Gang Wu, Run-Jie Wang, Yi Zhou, Xian-Yang Luo

**Affiliations:** ^1^ Department of Radiation Oncology, Xiamen Cancer Quality Control Center, Xiamen Cancer Center, Xiamen Key Laboratory of Radiation Oncology, the First Affiliated Hospital of Xiamen University, School of Medicine, Xiamen University, Xiamen, China; ^2^ Department of Otolaryngology-Head and Neck Surgery, Xiamen Key Laboratory of Otolaryngology-Head and Neck Surgery, the First Affiliated Hospital of Xiamen University, School of Medicine, Xiamen University, Xiamen, China

**Keywords:** hypopharyngeal cancer, neoadjuvant chemotherapy, immunotherapy, tumor response, laryngeal preservation

## Abstract

**Purpose:**

To evaluate the efficacy and laryngeal function preservation of neoadjuvant treatment with chemotherapy and immune checkpoint inhibitor for locally advanced hypopharyngeal cancer (LAHPC).

**Methods:**

We retrospectively collected LAHPC patients who were diagnosed between February 2022 and June 2023. The patients received a combination of chemotherapy and immune checkpoint inhibitors as the neoadjuvant therapy. The response to treatment, laryngeal function preservation rate, and short-term survival were assessed.

**Results:**

A total of 20 patients were included. Of these patients, 17 (85.0%) had stage IVA-B disease. Ten (50%) and four (20%) patients achieved pathological complete response (PCR) and major pathological response (MPR) to the primary tumor, respectively. In addition, 6 patients had incomplete pathological response (IPR). In the neck, 19 patients had node-positive disease before treatment, and only 5 patients (26.4%) had PCR to regional lymph nodes. Pathologically positive lymph nodes were still observed in 14 (73.6%) patients. Significant downgrading on narrow-band imaging assessment in primary tumors was associated with a higher probability of PCR or MPR than those with IPR (92.9% vs. 33.3%, P=0.014). The overall rate of laryngeal preservation was 95.0%. No severe perioperative complications or perioperative death were found. All patients completed the recommended postoperative radiotherapy/chemoradiotherapy. The median follow-up period was 12.1 months. The 1-year progression-free survival and overall survival were 94.1% and 92.9%, respectively. During the follow-up period, all 19 patients who underwent laryngeal preservation surgery had their laryngeal function preserved.

**Conclusion:**

The addition of an immune checkpoint inhibitor to neoadjuvant chemotherapy effectively preserves laryngeal function without increasing complications related to surgery and postoperative radiotherapy in LAHPC.

## Background

Hypopharyngeal cancer (HPC) is a relatively rare malignant tumor in the head and neck, with an estimated 6475 and 2314 new cases occurring annually in China and the United States, respectively (1). More than 80% of patients were diagnosed with locally advanced hypopharyngeal carcinoma (LAHPC), and 35-66% of them would develop disease recurrence after multimodal treatment (2–4). The 5-year overall survival (OS) rate of LAHPC was only around 22-30% and the survival rates were still unchanged during the past decades (5–7). The optimal therapeutic strategies remain controversial in LAHPC (8, 9). The potential damage of surgery to organ function and related surgical complications may affect treatment decisions (10). Several prospective studies have demonstrated that the implementation of neoadjuvant chemotherapy (NAC) followed by radiotherapy can serve as a viable approach for organ preservation without compromising OS rates (11, 12). However, real-world data have raised concerns that radiotherapy-based treatment may be detrimental to the OS of HPC patients (3, 5, 13, 14).

Immune checkpoint inhibitor (ICI) has been an important drug development in head and neck cancer (HNC) patients after cetuximab in the past two decades (15, 16). Immune-inflamed pattern (74%) is the predominant preexisting immune profile in HPC and served as an independent predictor of unfavorable prognosis, which indicates the potential benefit of immunotherapy in HPC (17). Several studies have shown a low effectiveness rate of NAC for HPC, with a complete response (CR) rate of 0-5% (18–20). Several previous studies have assessed the efficacy of neoadjuvant ICI in patients with HNC, while HPC accounts for a relatively small proportion of enrolled patients (0-30%) (21–24). In this study, we explore the effect of NAC combined with ICI on initial efficacy and laryngeal function preservation in LAHPC.

## Materials and methods

### Patients

We retrospectively included patients who were diagnosed with LAHPC between February 2022 and June 2023 in our institution. Patients who met the following criteria were included: 1) stage III-IV hypopharyngeal squamous cell carcinoma; 2) treated with NAC combined with ICI; 3) treated with surgery after neoadjuvant treatment. Patients without histologically or cytologically confirmed HPC or without primary lesions resection was excluded. The study received approval from the Ethics Committee of the First Affiliated Hospital of Xiamen University. All patients provided written informed consent before treatment.

### Variables

The following variables were included in the analysis: age, gender, smoking history, alcohol histology, TNM classification, combined positive score (CPS), chemotherapy regimen, ICI regimen, surgical procedure, response to neoadjuvant treatment, and toxic effects. To explore any potential correlation between PD-L1 expression and the efficacy of neoadjuvant therapy, CPS of 22C3 was used to describe the expression of PD-L1 in primary lesions before neoadjuvant therapy via immunohistochemical staining. In the evaluation of CPS, the positive rate of PD-L1 immunostaining in tumor cells and the positive rate of PD-L1 immunostaining in immune cells infiltrating the tumor were measured independently. CPS was determined by summing these two rates together.

### Treatment

The treatment strategies of patients were formulated based on a multidisciplinary team in our institution. During the neoadjuvant therapy period, the patients received ICI combined with paclitaxel (Albuminbound) 260mg/m^2^ and cisplatin 60 mg/m^2^ using a three-week treatment cycle. The ICI included camrelizumab, tislelizumab, pembrolizumab, or nivolumab. The decision-making of the administration of ICI was mainly according to physician-specific preference. Due to significant price differences among different ICIs, the selection of specific ICIs was mainly based on patient preferences.

Surgery was performed approximately 4 weeks following the completion of the last cycle of neoadjuvant treatment. Regardless of any regression of the lesion after neoadjuvant therapy, all patients underwent surgical resection. In cases where there was a complete response (CR) or partial response (PR) to the primary lesion, a pyriform sinus resection or posterior pharyngeal wall resection was performed using a low-temperature plasma knife. Total or partial laryngopharyngectomy was performed in those with stable disease (SD) or progressive disease (PD) after neoadjuvant treatment. The extent of the primary lesion in the hypopharynx was determined based on imaging examination. Similarly, the scope of surgical resection after neoadjuvant therapy was also determined by referring to imaging examinations. According to the imaging examination of lymph node status during HPC diagnosis, ipsilateral or bilateral neck lymph node dissection was performed after neoadjuvant therapy.

All patients underwent postoperative radiotherapy/chemoradiotherapy to the tumor bed and the **tumor**-draining **lymph** nodes. The prescribed dose was 60-66 gray (Gy)/32 fractions (f) to the tumor bed and 54-60Gy/32f to the neck. Platinum-based concurrent chemoradiotherapy was conducted for patients with extranodal invasion, multiple lymph node metastases, or positive margins.

### Assessment of response to neoadjuvant chemotherapy and immunotherapy

White light imaging endoscopy, narrow-band imaging (NBI), computed tomography (CT), or magnetic resonance (MR) imaging were conducted to assess the extent of their lesions before neoadjuvant treatment and before surgery in all patients.

The response to neoadjuvant therapy using radiological assessment was defined as CR, PR, SD, or PD using the Response Evaluation Criteria in Solid Tumors version 1.1 standard.

Currently, there is no globally recognized NBI classification for hypopharyngeal lesions (25). Overall, Type I was commonly observed in normal mucosa and cysts. Type II was mainly observed in cases of inflammation. Type III was mainly observed in cases of lymphoid hyperplasia. Type IV was mainly observed in cases of low-grade intraepithelial neoplasia or lymphoid hyperplasia with inflammation. Type Va was mainly observed in cases of high-grade intraepithelial neoplasia or carcinoma in situ. Type Vb and Vc were considered as invasive carcinoma.

Postoperative pathology was assessed by examining the remaining tumors in the resected samples. Pathological complete response (PCR) was defined as the absence of any remaining tumor tissue in both the primary site and metastatic lymph nodes. PCR evaluations were conducted separately for primary tumors and cervical lymph nodes. A major pathological response (MPR) was defined as the presence of fewer than 10% viable tumor cells in the primary lesion. An incomplete pathological response (IPR) was defined as the presence of 10% or more viable tumor cells in the primary lesion.

### Adverse reactions after treatment

Adverse reactions were evaluated based on the Common Terminology Criteria for Adverse Events V5.0. Follow-up data was collected through outpatient visits or telephone interviews. Additionally, electronic laryngoscopy and CT/MR scans of the head and neck were conducted for further examination.

### Statistical analyses

Categorical variables were compared using the Chi-square test or Fisher’s test. We utilized multivariable logistic regression to assess the independent predictors associated with PCR or MPR. Progression-free survival (PFS) and overall survival (OS) were calculated using Kaplan–Meier methods. Multivariable Cox proportional hazards regression was not conducted in this study due to the limited sample size. All statistical analyses were conducted by SPSS version 26.0 (IBM Corp., Armonk, NY, USA). P values <0.05 were considered statistically significant.

## Results

### Patient characteristic

A total of 20 patients were included with a median age of 59 years (range, 39-78) (**Table 1**). There were 3 (15.0%) and 17 (85.0%) patients who had stage III and IV disease, respectively. All patients had reported as Type VB-VC using NBI assessment before neoadjuvant therapy. Moreover, there were 13 (65.0%) patients had CPS <20, and 7 (35.0%) patients had CPS ≥20. All patients completed at least two cycles of neoadjuvant therapy. There were 18 patients (90%) received two cycles of neoadjuvant therapy, one patient (5%) received three cycles of neoadjuvant therapy due to the delay in the surgery caused by the COVID-19 pandemic, and one patient (5%) received four cycles of neoadjuvant therapy due to poor response to neoadjuvant therapy. Regarding ICIs, 8, 7, 4, and 1 patients treated with camrelizumab (40.0%), tislelizumab (35.0%), pembrolizumab (20.0%), and nivolumab (5.0%), respectively.

**Table 1:** Patient clinicopathological characteristics.

Variables	n (%)	IPR (n=6) (%)	PCR or MPR (n=14) (%)	P
Age (years)				
<65	14 (70)	5(83.3)	9 (64.3)	0.613
≥65	6 (30)	1 (16.7)	5 (35.7)	
Smoking histology				
No	4 (20)	2 (33.3)	2 (14.3)	0.549
Yes	16 (80)	4 (66.7)	12 (85.7)	
Alcohol histology				
No	4 (20)	3 (50.0)	1 (7.1)	0.061
Yes	16 (80)	3 (50.0)	13 (92.9)	
BMI (kg/m^2^)				
<18.5	3 (15)	0 (0)	3 (21.4)	0.521
≥18.5	17 (85)	6 (100)	11 (78.6)	
Tumor grade				
Well differentiated	0 (0)	0 (0)	0 (0)	0.613
Moderately differentiated	14 (70)	5 (83.3)	9 (64.3)	
Poorly differentiated;	6 (30)	1 (16.7)	5 (35.7)	
Tumor location				
Pyriform sinus	17 (85)	4 (66.7)	13 (92.9)	0.681
Posterior wall	2 (10)	2 (33.3)	0 (0.0)	
Postcricoid area	1 (5)	0 (0)	1 (7.1)	
Tumor stage				
T1-2	4 (20)	0 (0)	4 (28.6)	0.267
T3-4	16 (80)	6 (100)	10 (71.4)	
Nodal stage				
N0-1	4 (20)	0 (0)	4 (28.6)	0.267
N2-3	16 (80)	6 (100)	10 (71.4)	
TNM stage				
III	3 (15)	0 (0)	3 (21.4)	0.521
IV	17 (85)	6 (100)	11 (78.6)	
PD-1 inhibitor				
Tislelizumab	7 (35)	3 (50.0)	4 (28.6)	0.643
Camrelizumab	8 (40)	2 (33.3)	6 (42.9)	
Pembrolizumab+Nivolumab	5 (25)	1 (16.7)	4 (28.6)	
CPS				
0-19	13 (65)	6 (100)	7 (50.0)	0.051
≥20	7 (35)	0 (0)	7 (50.0)	
NBI after neoadjuvant therapy				
Type I-IV	—	2 (33.3)	13 (92.9)	0.014
Type V	—	4 (66.7)	1 (7.1)	

CPS, combined positive score; T, tumor; N, nodal; M, metastatic; PD-1, programmed death 1; BMI, body mass index; PCR, pathological complete response; MPR, major pathological response; IPR, incomplete pathological response; NBI, narrow-band imaging.

### Response to neoadjuvant treatment before surgery

**Figure 1:**
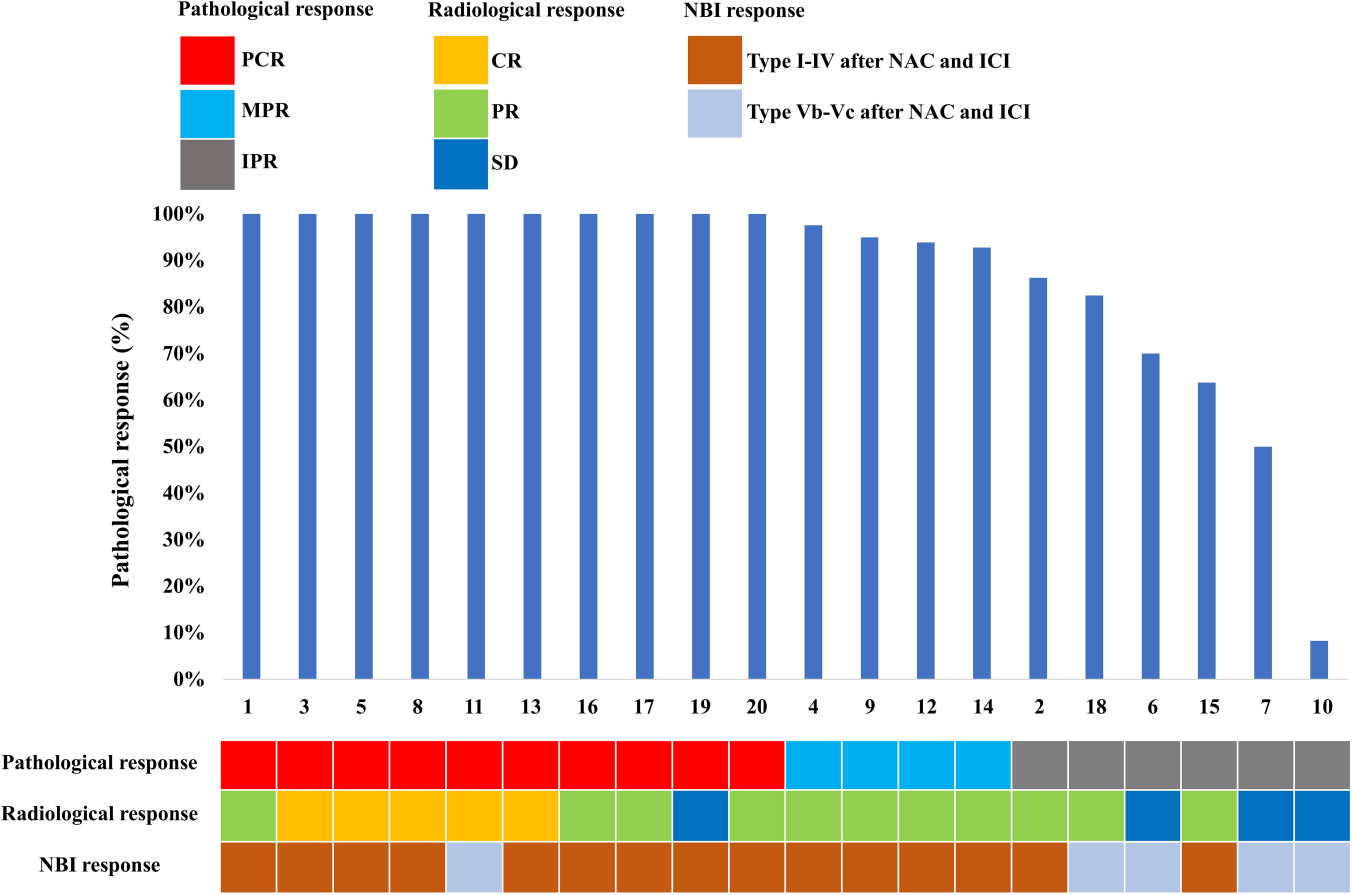
Treatment response of the study population.

Specific radiological and NBI responses after the completion of the NAC and ICI are shown in **Figure 1**. In terms of the primary tumor, 5 (25.0%) patients showed a CR, 11 (55.0%) patients showed a PR, 4 (20.0%) had an SD, and 0 (0%) had a PD. In the neck, 19 patients had node-positive disease and 15 (78.9%) had radiological nodes that persisted after neoadjuvant therapy. There were 4 (21.0%), 7 (36.8%), 7 (36.8%), and 1 (5.3%) had CR, PR, SD, and PD in the neck lymph nodes after neoadjuvant therapy.

Regarding the NBI assessment, there were 5 (25%) patients remained recorded as Type Vb or Vc after NAC and ICI. In addition, 15 (75%) patients had recorded as Type I-IV, including 9 (60%), 1 (6.7%), 2 (13.3%), and 3 (18.8%) patients recorded as Type I, II, III, and IV, respectively.

### Adverse reactions after neoadjuvant treatment

A total of 18 patients experienced treatment-associated adverse events (**Table 2**). Regarding hematological toxicity, 3 (15%), 2 (10%), 2 (10%), 2 (10%), and 1 (5%) patients experienced anemia, leukopenia, neutropenia, lymphocytopenia, and thrombocytopenia, respectively. We routinely used polyethylene glycol recombinant human granulocyte colony-stimulating factor for patients undergoing chemotherapy, thus there were no patients who had grade 3-4 myelosuppression.

**Table 2:** Acute Toxicities during neoadjuvant treatment with chemotherapy and an immune checkpoint inhibitor (n=20).

Toxicities	Grade 1-2 (%)	Grade 3	Grade 4
Hematologic			
Leukopenia	2 (10)	0	0
Neutropenia	2 (10)	0	0
Anemia	3 (15)	0	0
Thrombocytopenia	1 (5)	0	0
Lymphocytopenia	2 (10)	0	0
Nonhematologic			
Hepatotoxicity	2 (10)	0	0
Nephrotoxicity	0	0	0
Mucositis	0	0	0
Nausea	4 (20)	0	0
Vomiting	4 (20)	0	0
Rash	6 (30)	0	0
Fatigue	8 (40)	0	0
Neurotoxicity	9 (45)	0	0
Diarrhea	1 (5)	0	0
Thyroid dysfunction	4 (20)	0	0
Pruritus	0	0	0
Reactive cutaneous capillary endothelial proliferation *	2 (25)	0	0

*Eight patients received camrelizumab.

In terms of non-hematological toxicity, the most common were peripheral sensory neuropathy (n=9, 45.0%), fatigue (n=8, 40%), rash (n=6, 30.0%), thyroid dysfunction (n=4, 20%), nausea (n=4, 20%), and vomiting (n=4, 20.0%). No grade 3-5 adverse events were observed. Among the 8 patients who received treatment with camrelizumab, 2 patients (25%) experienced reactive cutaneous capillary endothelial proliferation, all of which were grade 1 or 2.

### Surgery procedures

The patients underwent surgery with an average interval of 30.5 days. Among those with CR or PR to the primary tumors, a pyriform sinus resection or posterior pharyngeal wall resection was performed (n=16 patients). In those with SD or PD to the primary tumor, three patients received partial laryngectomy and hypopharyngectomy and one patient received a total laryngectomy and hypopharyngectomy. The overall rate of laryngeal preservation was 95.0% (19/20). Ipsilateral and bilateral modified radical cervical lymph node dissection was performed in 15 (75.0%) and 5 patients (25.0%), respectively. No severe perioperative complications or perioperative deaths were found.

### Response to neoadjuvant treatment using pathological assessment

Specific pathological responses to NAC and ICI are shown in **Figure 1**. Ten patients (50%) and 4 (20%) patients achieved PCR and MPR to the primary tumor, respectively. In addition, 6 (30%) patients had IPR. In three patients with IPR, one presented with carcinoma *in situ* in surgical margin and 2 patients had positive surgical margins. One patient achieved PCR by the pathological assessment of the surgical specimen, but the radiological assessment was categorized as SD.

Regarding the regional lymph nodes, pathologically positive lymph nodes were found in 14 (73.6%) of 19 patients. Only 5 patients (26.4%) had PCR to the regional lymph nodes. One patient had node-negative using radiological assessment before NAC and ICI and also had node-negative after cervical lymph node dissection (30 lymph nodes on the ipsilateral neck were all negative).

### Response to neoadjuvant treatment between NBI and pathological assessment

**Figure 2:**
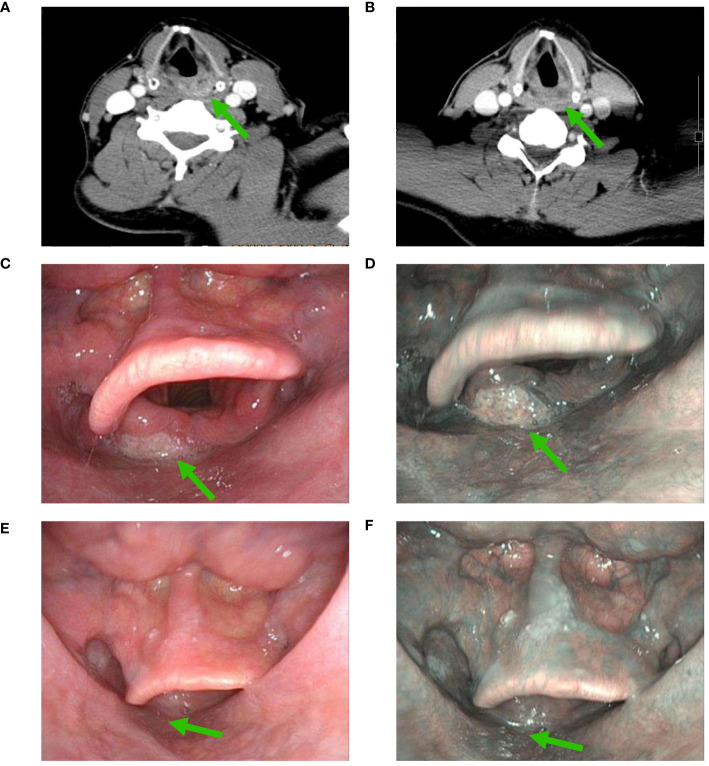
Changes in a patient with significant downgrading on NBI assessment after NAC and ICI **(A)**, CT scan before NAC and ICI; **(B)**, partial response using CT assessment after NAC and ICI; **(C)**, white light imaging assessment before NAC and ICI; **(D)**, NBI assessment before NAC and ICI; **(E)**, white light imaging assessment after NAC and ICI; **(F)**, a significant downgrading on NBI assessment after NAC and ICI (CT, computed tomography; NAC, neoadjuvant chemotherapy; ICI, immune checkpoint inhibitor; NBI, narrow band imaging) (The green arrow refers to the primary hypopharyngeal lesion).

**Figure 3:**
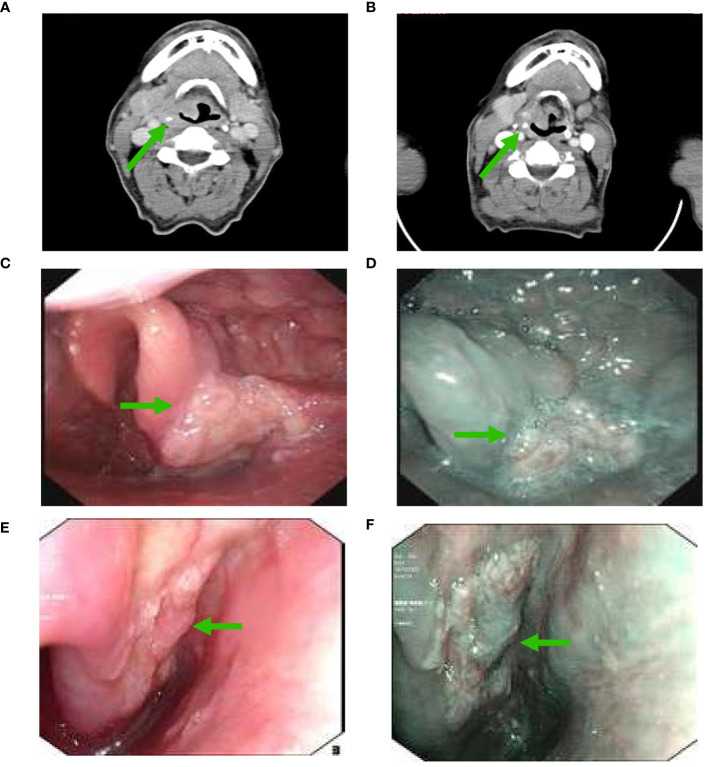
Changes in a patient with non-significant downgrading on NBI assessment after NAC and ICI **(A)**, CT scan before NAC and ICI; **(B)**, partial response using CT assessment after NAC and ICI; **(C)**, white light imaging assessment before NAC and ICI; **(D)**, NBI assessment before NAC and ICI; **(E)**, white light imaging assessment after NAC and ICI; **(F)**, a non-significant downgrading on NBI assessment after NAC and ICI (CT, computed tomography; NAC, neoadjuvant chemotherapy; ICI, immune checkpoint inhibitor; NBI, narrow band imaging) (The green arrow refers to the primary hypopharyngeal lesion).

In those with PCR to the primary tumor (n=10), 3 (30%), 1 (10%), 2 (20%), 2 (20%), and 1 (10%) patients showed Type I, II, III, IV, and IVB using NBI assessment, respectively (**Figures 1**, **2**). In those with MPR to the primary tumor (n=4), all patients had recorded as Type I using NBI assessment. However, in those with IPR (n=6), 2 (33.3%) and 4 (66.7%) patients were recorded as Type I and VB, respectively (**Figures 1**, **3**).

We found that significant downgrading on NBI assessment was associated with a higher probability of PCR or MPR (92.9% vs. 33.3%, P=0.014) (**Table 1**). There was no significant correlation between other variables and the PCR or MPR rate. The multivariable logistic regression confirmed that significant downgrading on NBI assessment was the independent predictor associated with PCR or MPR (odds ratio 0.035, 95% confidence interval 0.002-0.721, P=0.030).

### The completeness of postoperative radiotherapy

All patients received postoperative radiotherapy/chemoradiotherapy within six weeks after surgery. There were 14 (70%) patients received platinum-based concurrent chemoradiotherapy and 6 (30%) patients received radiotherapy alone. All patients completed the recommended radiotherapy/chemoradiotherapy, and three patients used a feeding tube due to grade 3 mucositis.

### Survival

**Figure 4:**
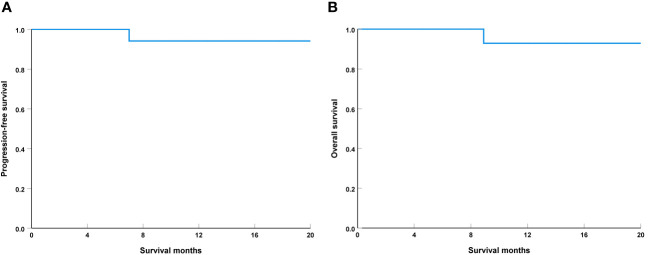
Progression-free survival **(A)** and overall survival **(B)** in the study population.

The median follow-up was 12.1 months (range, 4-20 months), one patient with IPR had local tumor recurrence in 7.0 months and died with this disease in 8.9 months. The 1-year PFS and OS were 94.1% and 92.9%, respectively (**Figure 4**). During the follow-up period, all 19 patients who underwent laryngeal preservation surgery had their laryngeal function preserved.

## Discussion

In this study, we explored the preliminary efficacy of combined IC and immunotherapy in preserving the laryngeal function of LAHPC. Our study found that the new induction therapeutic regime safely and effectively preserves laryngeal and swallowing function without increasing complications related to surgery and postoperative radiotherapy.

The immune-inflamed pattern is the predominant preexisting immune profile in HPC (17, 20), which suggests the potential benefit of immunotherapy in HPC. A recent prospective study included 15 LAHPC patients who received NAC combined with pembrolizumab. The overall rate of laryngeal preservation was 86.6%. After surgery, 4 had PCR (26.6%), 2 had MPR (13.3%), and 9 had IPR (60.0%) (26). A retrospective study included 156 patients with locally advanced laryngeal and hypopharyngeal squamous cell carcinoma treated with PD-1 inhibitors combined with NAC (48.7% of HPC), the results found that 23.1%, 65.4%, 9.0%, and 3.0% of patients demonstrated a radiological CR, PR, SD, and PD, respectively. However, only 26 patients (16.7%) underwent surgical treatment, therefore, it was not possible to accurately assess the pathological response after neoadjuvant treatment (27). In our study, we found a PCR rate of 50% and an MPR rate of 20% after neoadjuvant treatment. The PCR rates of NAC plus ICI based on the current literature including ours have exceeded the previous combination chemotherapy regimens (17–19).

In HPC, the laryngeal preservation rate and laryngeal function preservation rate could be improved using NAC (28), but it does not have a significant impact on OS (29). Moreover, currently, data on the impact of tumor response after neoadjuvant immunotherapy on survival are still immature (30). A previous study from other HNC showed that patients who achieve an MPR after neoadjuvant immunotherapy have a 2-year progression-free survival rate of 100%, significantly better than patients with an IPR (23). Therefore, more data accumulation and longer follow-up should be undertaken to explore the impact of tumor response on the long-term survival of LAHPC patients after NAC plus immunotherapy.

Conventional tumor assessment criteria may not be sufficient to accurately evaluate the tumor response in the era of immunotherapy. The study by Wang et al. showed no patients reached a radiological CR but the postoperative evaluation showed a PCR rate of 26.6% after NAC and ICI (26). Therefore, accurate assessment of tumor response is crucial for subsequent treatment decisions in the era of immunotherapy. In our study, we employed NBI to evaluate the response of primary tumors. We found that 92.9% (13/14) of patients with PCR or MPR to the primary tumor showed significant downgrading on NBI assessment. The study by Lu et al. found that NBI had significantly higher diagnostic accuracy and sensitivity for hypopharyngeal lesions than white light imaging endoscopy (31). The radiological assessment primarily evaluates the size of the tumor and the NBI allows us to observe the microvascular patterns and invasion depth of the superficial carcinoma (32). Based on our findings, NBI may have a certain supplementary value for evaluating tumor response after NAC and immunotherapy. However, NBI can only observe the superficial distribution of tumor blood vessels, making it challenging to accurately assess tumors with deep invasion.

Due to the potential complications and functional disorders after surgery (10), the majority of patients in the real world tend to opt for definitive chemoradiotherapy (5). Nevertheless, definitive chemoradiotherapy may have higher recurrence rates and inferior OS rates compared to surgical treatment (5, 13, 14). In this study, patients with CR or PR to primary tumor underwent a local extended resection, thus we could accurately assess the pathological remission of tumors and minimize damage to laryngeal function.

Several studies have found that metastatic neck nodes were often less responsive to NAC, and recurrence could occur in only regional sites (33, 34). In this study, we found differences in the efficacy of NAC and ICI on PCR rate between primary lesions (50.0%) and metastatic neck lymph nodes (26.4%). Fang et al. also showed a lower response rate in cervical lymph node metastasis of locally advanced laryngeal and hypopharyngeal cancers using NAC and ICI (27), which suggests that this subset of patients may benefit from additional treatment considerations. The reasons for the lower response of neck lymph nodes compared to the primary lesion remain unknown. In the study of squamous cell carcinoma of the oropharynx, a higher infiltration of Tregs in metastatic lymph nodes was found and can be a potential driver of an immunosuppressive milieu leading to favor cancer progression (35). In tumor immunity, patients with high expression of Treg show lower sensitivity to immunotherapy as they suppress immune responses (36). Rahim et al. further found that in cases of human HNC, the dynamic CD8+ T cell responses to immunotherapy in regional lymph nodes were impaired in metastatic lymph nodes (37). These findings provide a basis for the potential advancement of immunotherapy that effectively utilizes anti-tumor immunity in human lymph nodes and contributes to the development of immune-monitoring approaches for cancer patients undergoing immunotherapy.

Several studies have reported that there is no significant correlation between the expression of PD-L1 and the prognosis of HPC (18, 38). However, the relationship between PD-L1 expression levels and the response to neoadjuvant therapy remains uncertain. Previous studies have shown no significant correlation between PD-L1 expression and the response to NAC, but it is important to note that those studies did not include patients treated with ICI (18). Conversely, Wang et al. observed that a CPS >5 in the biopsies of primary lesions was associated with a higher rate of PCR (26). In our study, we found no correlation between PCR or MPR and CPS. Given the limited research on neoadjuvant ICI in HPC and the small sample size in our study, it is necessary to accumulate more data in the future to identify predictive biomarkers for immunotherapy in HPC.

In this study, all patients underwent postoperative radiotherapy/chemoradiotherapy, and no additional adverse reactions during radiotherapy/chemoradiotherapy were found. We did not incorporate ICI during postoperative radiotherapy/chemoradiotherapy because several prospective studies have found that adding ICI during radiotherapy/chemoradiotherapy did not improve survival in patients with locally advanced HNC (39, 40). However, an area of concern is whether patients who achieve PCR in the primary lesion after NAC and ICI still require tumor bed radiotherapy in addition to surgical resection.

Several limitations should be acknowledged in our study. First, our study was a retrospective analysis, which can introduce inherent biases and limit the level of evidence. Second, we only included a limited sample size of patients in this study and were unable to precisely analyze the factors associated with PCR after neoadjuvant therapy, which could reduce the statistical power and generalizability. Third, the duration of follow-up is another important limitation in this study. Moreover, the toxicities of neoadjuvant chemotherapy and ICI treatment tend to be underestimated in retrospective studies. Finally, the swallowing and speech functions as well as quality of life were not assessed in this study.

## Conclusions

In conclusion, our study suggests that the addition of ICI to NAC effectively preserves laryngeal function without increasing complications related to surgery and postoperative radiotherapy in LAHPC. Prospective randomized controlled trials are required to confirm our findings and establish the role of neoadjuvant ICI with chemotherapy in the laryngeal function preservation and long-term survival of LAHPC patients.

## Data availability statement

The raw data supporting the conclusions of this article will be made available by the authors, without undue reservation.

## Ethics statement

The studies involving humans were approved by the Ethics Committee of the First Affiliated Hospital of Xiamen University. All patients provided written informed consent before treatment. The studies were conducted in accordance with the local legislation and institutional requirements. Written informed consent for participation in this study was provided by the participants’ legal guardians/next of kin.

## Author contributions

SW: Data curation, Formal Analysis, Writing – original draft. RW: Conceptualization, Data curation, Formal Analysis, Investigation, Writing – original draft. YZ: Software, Supervision, Visualization, Writing – review & editing. XL: Software, Supervision, Validation, Visualization, Writing – review & editing.
